# Stress and hope distinguish individuals with suicidal plan from suicide ideators among Chinese college students

**DOI:** 10.3389/fpsyt.2024.1387868

**Published:** 2024-08-09

**Authors:** Naiche Chen, Zheru Dai, Yongguang Wang

**Affiliations:** ^1^ Ideological and Political Education Center, Northeast Normal University, Changchun, Jilin, China; ^2^ Mental Health Education Center, Wenzhou Medical University, Wenzhou, Zhejiang, China; ^3^ Affiliated Mental Health Center & Hangzhou Seventh People’s Hospital, Zhejiang University School of Medicine, Hangzhou, Zhejiang, China

**Keywords:** suicidal ideation, suicide plan, stress, hope, college students, ideation-to-action framework

## Abstract

**Background:**

Suicide among college students is a significant public health concern in China. While suicidal ideation serves as a crucial predictor of subsequent suicidal plans and behaviors, it is important to recognize that most instances of suicidal ideation may only be fleeting thoughts that do not progress to an actual plan. Therefore, it is imperative to identify the factors associated with the transition from suicidal ideation to a concrete plan. Consequently, this study aims to investigate whether certain frequently cited factors can differentiate individuals who have formulated a specific suicidal plan from those who have experienced suicidal thoughts without planning, based on data obtained through a cross-sectional survey.

**Materials and methods:**

This survey was conducted as part of routine mental health assessments among second-year college students in October 2023. Data from a total of 4,858 second-year college students were utilized for the final analyses. Two survey questions were employed to identify past-year suicidal ideation and past-year suicidal plan. All participants were required to complete various assessments, including the Chinese version of Depression Anxiety Stress Scale 21 items (DASS-21), the Chinese version of Gratitude Questionnaire-six items (GQ-6), the Chinese version of Meaning in Life Questionnaire (MLQ), and the Chinese version of State Hope Scale (SHS).

**Results:**

Among 4,858 participants, a total of 134 individuals (2.8%) were confirmed to have experienced past-year suicidal ideation. Out of these, 53 (1.1% overall) reported having a past-year suicidal plan, accounting for approximately 39.6% of those with suicidal ideation. Logistic regression analyses revealed that while most potential variables differentiated between students with and without suicidal ideation, only two factors stood out in distinguishing individuals with a suicidal plan from those who had not made such plans despite experiencing suicidal thoughts—presence of stress (*OR*=2.49, *95% CI*: 1.04–5.96) and lower scores of hope agency (*OR*=0.84, *95% CI*: 0.72–0.98).

**Conclusion:**

These findings suggest that the stress may contribute to susceptibility for transitioning from mere thoughts to actual planning regarding suicide; conversely, hope agency appears to offer protection against this transition process. Therefore, we advocate for targeted interventions aimed at fostering hope among individuals who have encountered adverse and stressful life events.

## Introduction

Suicide ranks as one of the primary causes contributing to morbidity and mortality among young adults globally. In China, specifically, it is estimated that approximately 10.7% of college students have experienced suicidal ideation (1) and 4.4% have formulated plans toward such ends (2). While there exists no precise national data regarding suicide rates among this population at the present time, suicide among college students presents a significant public health issue throughout China. Although contemplating suicide serves as an important indicator for predicting future attempts or completed acts thereof, many instances where individuals experience these thoughts are transient phenomena which do not necessarily culminate into concrete plans or actions; thus, identifying variables which contribute toward transitioning from mere contemplations toward actualized planning remains crucially important (3–6).

To date, numerous studies have been conducted to investigate the factors associated with suicidal ideation among Chinese college students. The presence of psychiatric symptoms has consistently emerged as the most reliable risk factor for suicidal ideation. For example, meta-analytic data revealed that depressive symptoms were associated with a 3.2-fold increase in the likelihood of suicidal ideation among Chinese college students (7). Similarly, a meta-analysis involving 66,752 participants reported a strong association between anxiety symptom and students’ suicidal ideation (8). Additionally, stress symptoms have been identified as predictors of suicidal ideation (9). It is estimated that approximately 20% of patients with schizophrenia attempt suicide at least once during their life (10). In addition, exposure to adverse childhood experiences strongly increases the probability of suicide behaviors in patients with schizophrenia (11). However, it remains unknown whether these psychiatric symptoms can predict the occurrence of suicidal plans among individuals experiencing suicidal thoughts.

On the contrary, in line with a positive psychology framework, numerous psychological variables have been proposed as potential protective factors against suicide. For instance, accumulating evidence suggests that gratitude is linked to reduced psychological distress (12) and decreased overall symptoms of psychopathology (13), thereby conferring resilience against suicide (14). Furthermore, a correlation has been found between the meaning of life and lower levels of suicidal ideation and attempts (15). Additionally, hope has consistently been reported to be associated with greater life satisfaction, reduced psychological distress, and enhanced psychological well-beings, thus acting as a buffer against suicide (16). Again, it remains unclear whether these positive psychological variables can effectively prevent the transition from ideation to an actual plan.

According to the ideation-to-action framework (3–6), it is crucial to identify variables that can effectively predict and elucidate the transition from suicidal ideation to a concrete suicidal plan. Given that virtually all students with a suicidal plan also experience suicidal ideation, it becomes imperative to control for this factor when attempting to identify predictors of a suicide plan. However, as far as our knowledge extends, no studies have thus far examined the factors that predict a suicide plan among Chinese college students who have experienced suicidal thoughts. This research gap is significant because gaining a better understanding of the progression from suicidal ideation to planning can greatly facilitate the development of targeted intervention programs for individuals at risk. Consequently, this study aims to investigate whether certain frequently cited factors can differentiate between those who have formulated a specific suicide plan and those who have only experienced suicidal thoughts without any planning, based on data obtained through a cross-sectional survey.

## Methods

### Sampling and participants

This survey was conducted as part of routine mental health assessments among second-year college students in October 2023. The current study was approved by the Ethics Committee of Wenzhou Medical University (Ethics Approval Code: 2023–021). Informed consent was obtained from all subjects. In total, 5,652 second-year college students should have participated in the survey, and actually 5,174 students completed the assessments (i.e., survey response rate was 91.5%). A total of 316 participants gave exactly the same option on each questionnaire/scale, so these data were defined as unqualified data and excluded from the analysis. Finally, data from 4,858 participants (1,893 men and 2,965 women, mean age was 19.32 years) were utilized for the final analyses.

### Measures

#### Past-year suicidal ideation and past-year suicidal plan

Two survey questions were employed to identify past-year suicidal ideation and past-year suicidal plan. All participants were first asked, “In the past 12 months, did you seriously think about trying to kill yourself?” Then, those who responded “YES” were further asked, “During the past 12 months, did you make any plans to kill yourself?”

**Figure 1:**
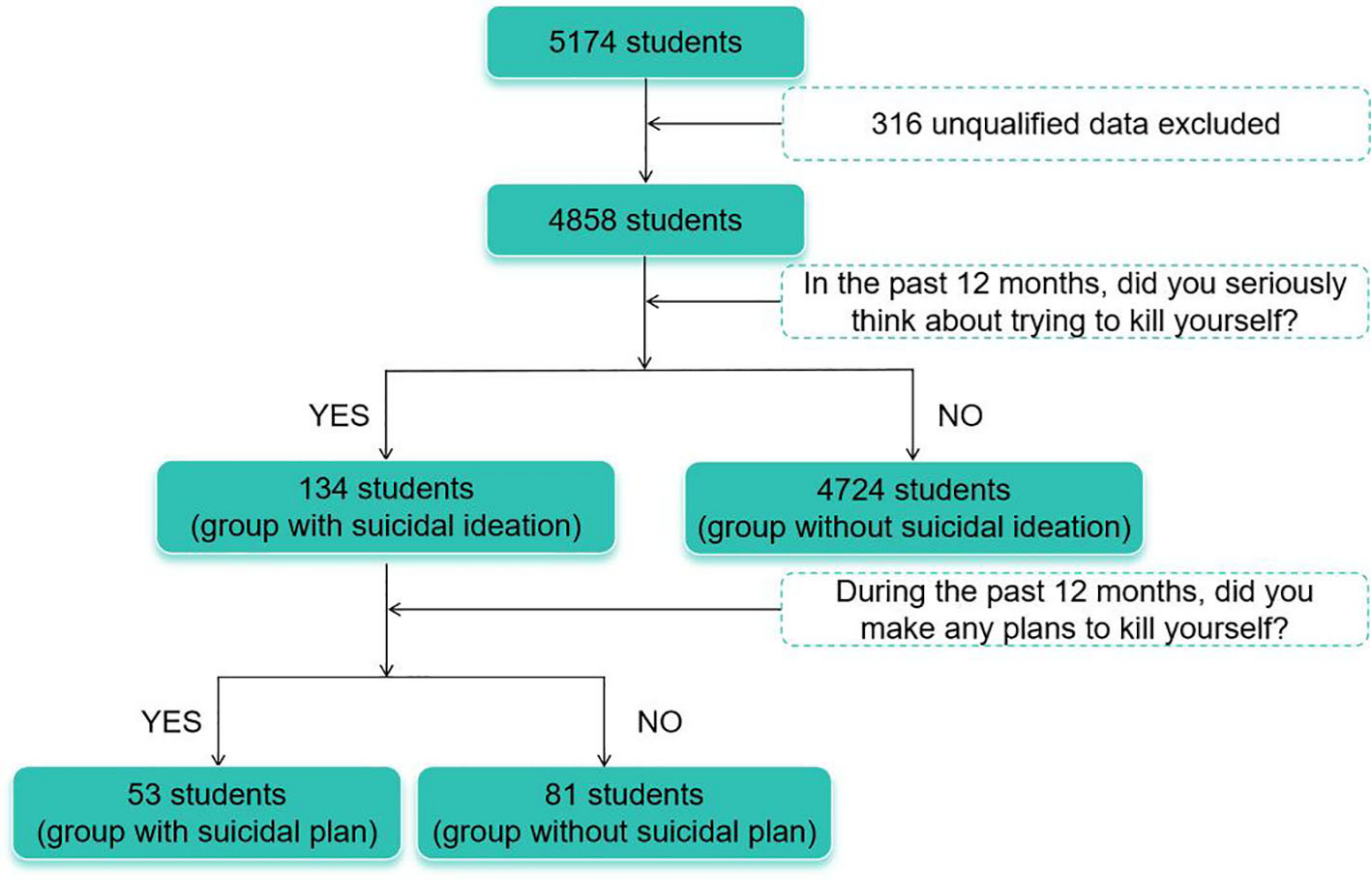
Sampling profile.

As **Figure 1** shows, 134 participants were categorized as group with suicidal ideation. Among these with suicidal ideation, 53 participants had suicidal plan (i.e., group with suicidal plan).

#### Depression, anxiety, and stress

The presence of symptoms of depression, anxiety, and stress were measured with the Chinese version of Depression Anxiety Stress Scale 21 items (DASS-21) (17, 18). DASS-21 is a self-report measure of mental health status, including depression, anxiety, and stress severity. The total scores for each subscale range from 0 to 42, with higher scores indicating higher levels of depression, anxiety, and stress. In the present study, cutoff scores of ≥10, ≥8, and ≥15 were adopted to define the presence symptoms of depression, anxiety, and stress, respectively (19). The Cronbach’s alpha for the DASS-21 in the present study is 0.886 (depression), 0.838 (anxiety), 0.883 (stress), and 0.949 (total scores), separately.

#### Gratitude

Gratitude was measured with the Chinese version of Gratitude Questionnaire-six items (GQ-6) (14, 20). The GQ-6 contains six items, with each item scored from 1 (strongly disagree) to 7 (strongly agree). The total scores of the GQ-6 range from 7 to 42, with higher scores indicating greater gratitude. The Cronbach’s alpha for the GQ-6 in the present study is 0.724.

#### Meaning in life

Meaning in life was measured with the Chinese version of Meaning in Life Questionnaire (MLQ) (21, 22), which measures presence of meaning and search for meaning of life. The Chinese version of MLQ contains nine items, with each item scored from 1 (strongly disagree) to 7 (strongly agree). The total scores of the subscale for presence of meaning in life (MLQ-P) range from 7 to 35, with higher scores indicating higher level of presence of meaning of life. The total scores of the subscale for search of meaning in life (MLQ-L) range from 7 to 28, with higher scores indicating higher level of active search for meaning in life. The Cronbach’s alpha for the MLQ in the present study is 0.907 (MLQ-P) and 0.891 (MLQ-S), separately.

#### Hope

The hope level was measured by the Chinese version of State Hope Scale (SHS; 23, 24), which measures beliefs about how successful in pursuing goals (Agency) and how confident in finding ways to attain goals (Pathways). The Chinese version of SHS contains six items, with each item scored from 1 (definitely false) to 8 (definitely true). The total scores of the subscale for Agency (SHS-A) and the subscale for Pathways (SHS-P) both range from 8 to 24, with higher scores representing a heightened sense of perceived hope. The Cronbach’s alpha for the SHS in the present study are 0.912 (SHS-A) and 0.947 (SHS-P), separately.

### Analysis procedure

Statistical analyses were performed using the SPSS statistical package version 17.0. Chi-square test and one-way ANOVA were used to compare between groups (i.e., Group with suicidal ideation vs. Group without suicidal ideation; Group with suicidal plan vs. Group without suicidal plan but with suicidal ideation) for categorical variables and continuous variables, separately. Logistic regression was conducted to assess factors associated with suicidal ideation and suicidal plan, adjusting for gender and age. Specifically, logistic regression was first conducted to examine factors associated with suicidal ideation among all respondents. Then, we conducted the logistic regression to analyze what distinguished individuals with suicidal plan and from those without suicidal plan among students with suicidal ideation. For DASS-21, only the presence of depression, anxiety, and stress were entered into the logistic regression model.

## Results

### Group comparisons

#### Comparisons between groups with or without suicidal ideation

As **Figure 1** shows, out of 4,858, 134 (2.8%) were confirmed as individuals with past-year suicidal ideation. **Table 1** summarizes the details of group comparisons between group with suicidal ideation and group without ideation. As **Table 1** shows, the group with suicidal ideation had significantly higher prevalence of depression, anxiety, and stress than the group without suicidal ideation (all *p*<0.01). One-way ANOVA indicated that the group with suicidal ideation had a significantly higher score on depression, anxiety, stress, GQ-6, MLQ-O, MLQ-S, SHS-A, and SHS-P (all *p* ≤ 0.01).

**Table 1:** Comparisons between groups with or without suicidal ideation.

	Group with suicidal ideation	Group without suicidal ideation	Statistics
	(n=4,724)	(n=134)	
Age (years)	19.32 ± 0.66	19.29 ± 0.72	*F*=0.21, *P*=0.65
Gender (female)	61.1% (2885)	59.7% (80)	χ^2^ = 0.10, *P*=0.75
DASS-21			
Presence of depression	13.4% (634)	60.4% (81)	χ2 = 229.59, *P*<0.01
Presence of anxiety	20.2% (954)	64.2% (86)	χ^2^ = 149.83, P<0.01
Presence of stress	8.4% (398)	39.6% (53)	χ2 = 149.91, *P*<0.01
Total scores of depression	3.17 ± 5.21	13.00 ± 9.80	*F*=433.32, *P*<0.01
Total scores of anxiety	3.73 ± 5.16	11.78 ± 8.47	*F*=303.11, *P*<0.01
Total scores of stress	5.31 ± 6.59	14.48 ± 8.87	*F*=246.36, *P*<0.01
Total scores of GQ-6	33.86 ± 5.77	30.81 ± 6.51	*F*=35.91, *P*<0.01
MLQ			
Total scores of MLQ-P	25.51 ± 6.00	20.73 ± 7.21	*F*=81.71, *P*<0.01
Total scores of MLQ-S	22.15 ± 4.37	21.19 ± 4.59	*F*=6.22, *P*=0.01
SHS			
Total scores of SHS-A	17.80 ± 4.00	13.59 ± 4.34	*F*=143.96, *P*<0.01
Total scores of SHS-P	18.33 ± 3.80	15.01 ± 4.26	*F*=98.84, *P<*0.01

#### Comparisons between group with suicidal plan and group without suicidal plan but with suicidal ideation

As shown in **Figure 1**, 53 (1.1%) of the 4,858 students reported having past-year suicidal plan, representing 39.6% of the students with suicidal ideation. **Table 2** summarizes the details of comparisons between group with suicidal plan and group without suicidal plan but with suicidal ideation. As **Table 2** shows, the group with suicidal plan had significantly higher prevalence of stress than the group without suicidal plan but with suicidal ideation (*p*<0.01). One-way ANOVA indicated that the group with suicidal plan had a significantly higher score on SHS-A (all *p*=0.03). The difference between groups on total scores of depression and total scores of stress approached significance (both *p*=0.05). No significant difference was found for the prevalence of depression, anxiety, scores on GQ-6, MLQ-O, MLQ-S, and SHS-P between subgroups (all *p*≥0.28).

**Table 2:** Comparisons between subgroups with or without suicidal plan among students with suicidal ideation.

	Group with suicidal plan	Group without suicidal plan but with suicidal ideation	Statistics
	(n=53)	(n=81)	
Age (years)	19.40 ± 0.69	19.22 ± 0.74	*F*=1.86, *P*=0.17
Gender (female)	56.6% (30)	61.7% (50)	χ2 = 0.35, *P*=0.55
DASS-21			
Presence of depression	66.0% (35)	56.8% (46)	χ2 = 1.15, *P*=0.28
Presence of anxiety	67.9% (36)	61.7% (50)	χ^2^ = 0.54, P=0.47
Presence of stress	54.7% (29)	29.6% (24)	χ2 = 8.43, *P*<0.01
Total scores of depression	15.06 ± 11.55	11.65 ± 8.26	*F*=3.95, *P*=0.05
Total scores of anxiety	12.87 ± 9.24	11.06 ± 7.90	*F*=1.46, *P*=0.23
Total scores of stress	16.30 ± 10.79	13.28 ± 7.19	*F*=3.78, *P*=0.05
Total scores of GQ-6	29.91 ± 6.22	31.41 ± 6.67	*F*=1.71, *P*=0.19
MLQ			
Total scores of MLQ-P	21.23 ± 7.42	20.41 ± 7.09	*F*=0.41, *P=*0.52
Total scores of MLQ-S	20.87 ± 5.20	21.41 ± 4.16	*F*=0.44, *P*=0.51
SHS			
Total scores of SHS-A	12.58 ± 4.29	14.25 ± 4.28	*F*=4.83, *P*=0.03
Total scores of SHS-P	14.79 ± 4.36	15.15 ± 4.22	*F*=0.22, *P=*0.64

### Logistic regression equations

The results of logistic regression equations for suicidal ideation and suicide plan are shown in **Table 3**. Logistic regression analyses indicate that the presence of depression, anxiety, stress, and total scores of SHS-A significantly differentiated suicidal ideation from all other participants. For suicide plan, logistic regression analysis yielded a very different model, with only presence of stress and total scores of SHS-A significantly distinguishing between individuals with suicidal plan and those without suicidal plan who had suicidal ideation.

**Table 3:** Logistic regression analysis for suicidal ideation and suicide plan.

	For suicidal ideation*		For suicidal plan* (among individuals with suicidal ideation)	
	*OR (95% CI)*	*P*	*OR (95% CI)*	*P*
DASS-21				
Presence of depression	2.73 (1.61–4.64)	<0.01	0.93 (0.35–2.50)	0.89
Presence of anxiety	1.77 (1.05–2.97)	0.03	0.81 (0.31–2.11)	0.67
Presence of stress	1.65 (1.05–2.57)	0.03	2.49 (1.04–5.96)	0.04
Total scores of GQ-6	0.97 (0.94–1.00)	0.06	0.96 (0.90–1.03)	0.24
MLQ				
Total scores of MLQ-P	0.98 (0.95–1.02)	0.34	1.05 (0.99–1.12)	0.11
Total scores of MLQ-S	1.03 (0.99–1.08)	0.18	0.99 (0.90–1.08)	0.79
SHS				
Total scores of SHS-A	0.82 (0.76–0.89)	<0.01	0.84 (0.72–0.98)	0.03
Total scores of SHS-P	1.07 (0.98–1.16)	0.12	1.10 (0.95–1.27)	0.20

*, with adjusting for gender and age.

## Discussion

Suicide among college students remain a significant public health concern in China. In order to advance the prevention efforts, it is crucial to gain a deeper understanding of the factors that contribute to or deter the transition from suicidal ideation to actual plans. This study, aligned with an ideation-to-action framework (3–6), aimed to investigate whether certain frequently mentioned factors could differentiate individuals who had made specific plans for suicide from those who had experienced suicidal ideation without planning. Our primary findings indicate that, while most of these potential variables distinguished between students with and without suicidal ideation, only two factors (namely, presence of stress and lower scores of SHS-A) were able to distinguish individuals with concrete suicidal plans from those who had contemplated suicide but did not have a plan in place. These findings hold significant implications for the development of effective strategies for suicide prevention and intervention.

Regarding psychiatric symptoms, our findings suggest that depression and anxiety are significant predictors of suicidal ideation but do not differentiate between individuals with a suicidal plan and those without one. This pattern aligns with previous literature. Our previous study in college students demonstrated that screening positive for psychiatric disorders offered limited discriminatory value in distinguishing suicide attempters from suicidal ideators (25). Similarly, a meta-analysis of 27 studies revealed that while depression strongly predicted suicidal ideation, its effect was minimal to small when comparing attempters to ideators (4). Importantly, our results indicate a significant positive association between the presence of stress and the development of a suicidal plan among ideators. We cautiously speculate that stress symptoms may facilitate the transition from mere ideation to planning, as prior research has shown a correlation between perceived stress and increased likelihood of developing a specific suicidal plan one year later (26).

Our findings, in line with a positive psychology framework, revealed that individuals with suicidal ideation displayed significantly lower scores on GQ-6, MLQ-P, MLQ-S, SHS-P, and SHS-A compared with those without such thoughts. However, except for the scores of SHS-A, these positive psychological variables failed to distinguish between individuals with suicidal plans and those with ideation alone. These results suggest that hope agency may be the sole factor that provides protection against the transition from ideation to planning suicide. These findings are particularly intriguing and significant because hope agency could serve as a valuable target for interventions aimed at reducing suicide risk. Indeed, hope agency fosters a sense of connectedness to successful determination. According to the three-step theory (27), this connectedness can surpass their psychological pain or hopelessness, thereby impeding the progression from occasional suicidal desires to an actual plan.

Several limitations of the current study should be acknowledged. Firstly, a cross-sectional research design was employed instead of a longitudinal approach, thus warranting the need for future longitudinal and prospective studies to establish causal relationships among the variables. Secondly, the sample size of participants with suicidal plan was small, which limited statistical power in detecting differences. Thirdly, various confounding factors such as social support (28), perceived discrimination (29), gender identity, and sexual orientation may have influenced the results of this study. Therefore, future research design should incorporate additional relevant variables to enhance our understanding of the transition process from suicidal ideation to suicide planning. Lastly, potential issues like intentional non-reporting of suicidality and recall bias cannot be ruled out.

In conclusion, these findings suggest that the stress may contribute to vulnerability in transitioning from mere thoughts to actual planning regarding suicide; conversely, hope agency appears to offer protection against this transition process. Henceforth, special attention should be given by suicide prevention programs toward recognizing stress symptoms and implementing training programs for management of stress symptoms among students reporting suicidal ideation (30). Additionally, targeted interventions aimed at fostering hope among individuals who have experienced adverse and stressful life events are strongly advocated (31).

## Data Availability

The raw data supporting the conclusions of this article will be made available by the authors, without undue reservation.
